# Immunohistochemical, histopathological study and chemoprotective effect of *Solanum nigrum* in *N*-nitrosodiethylamine-induced hepatocellular carcinoma in Wistar rats

**DOI:** 10.14202/vetworld.2018.402-409

**Published:** 2018-04-04

**Authors:** G. M. Akshatha, S. K. Raval, G. M. Arpitha, S. H. Raval, D. J. Ghodasara

**Affiliations:** 1 Department of Veterinary Medicine, College of Veterinary Sciences and Animal Husbandry, Anand Agricultural University, Anand, Gujarat, India; 2 Department of Veterinary Parasitology, Madras Veterinary College, Tamil Nadu Veterinary and Animal Sciences University, Chennai, Tamil Nadu, India; 3 Department of Veterinary Pathology, College of Veterinary Sciences and Animal Husbandry, Sardarkrushinagar Dantiwada University, Dantiwada, Gujarat, India; 4 Department of Veterinary Pathology, College of Veterinary Sciences and Animal Husbandry, Anand Agricultural University, Anand, Gujarat, India

**Keywords:** hepatocellular carcinoma, histopathology, immunohistochemistry, *Solanum nigrum*

## Abstract

**Background and Aim:**

Cancer is a devastating disease with a severe impact on the physical and psychological well-being of patients. Hepatocellular carcinoma (HCC) has been reported in various species of animals including dogs, cats, sheep, and pigs. The present study aimed to study the immunohistochemical and histopathological changes andchemoprotective effect of aqueous and alcoholic extracts of *Solanum nigrum* on *N*-nitrosodiethylamine (NDEA)-induced HCC rat model.

**Materials and Methods:**

Eighty-two male Wistar rats of 15 weeks of age weighing 200-250 g were selected for the experiment. They were randomly divided into ten groups. Group I served as normal control consisted of healthy rats. HCC was induced in Group II, IV, V, VI, VII, and X rats using NDEA as inducing agent followed by phenobarbitone as a promoter for 16 weeks. Group II rats were kept untreated as HCC control. Group III rats were kept as vehicle control (0.05% Sodium bicarbonate). Group IV and V rats were treated with aqueous extract of *S. nigrum* at 200 mg/kg and 400 mg/kg, respectively, and Group VI and VII rats were treated with an alcoholic extract of *S. nigrum* at 200 mg/kg and 400 mg/kg, respectively, daily orally for 28 days. Group X rats were treated withsorafenib as reference drug at a dose of 11.4 mg/kg daily orally for 28 days. Group VIII and IX rats were kept as aqueous and alcoholic extract control for studying the effect of the same on normal rats. Liver samples were collected to study the gross and histopathological lesions and the activity of cleaved caspase-3 and chemopreventive effect of aqueous and alcoholic extracts of *S. nigrum* on HCC.

**Results:**

The liver sections of rats from HCC control (Group II) showed loss of lobular architecture, necrosis, fatty change, enlarged and darkened nuclei with variable size, dilatation of hepatic sinusoids with Kupffer cell hyperplasia, dilatation and proliferation of bile duct, and intranuclear vacuoles and also showed the presence of more than one nucleolus. Administration of alcoholic extract of *S. nigrum* and sorafenib to NDEA/phenobarbital-treated rats reduced the severity of lesions in the liver. Immunohistochemical analysis of liver sections for caspase-3-positive cells of hepatic cancer-induced group showed immunoreactivity to rarely few. The immunoreactivity of the hepatocytes treated with a higher dose of alcoholic extract of *S. nigrum* was limited and was comparable to a standard drug, sorafenib.

**Conclusion:**

Oral administration of aqueous and alcoholic extracts of *S. nigrum* for 28 days showed significant rejuvenation in the structure of the liver in the histopathological section in a dose-dependent manner in rats.

## Introduction

*N*-nitrosodiethylamine (NDEA) also known as diethylnitrosamine (DEN), a hepatocarcinogen, is known to cause perturbations in the nuclear enzymes involved in deoxyribonucleic acid (DNA) repair/replication and is normally used as a carcinogen to induce liver cancer in animal models [1]. DEN has been shown to be metabolized to its active ethyl radical metabolite, and the reactive product interacts with DNA causing mutation, which would lead to carcinogenesis [2,3]. Experimental, clinical, and epidemiological studies have provided evidence supporting the role of reactive oxygen species in the etiology of cancer. DEN has been suggested to cause oxidative stress and cellular injury due to the enhanced formation of free radicals [4,5].

Recent approach of chemoprevention serves as an attractive alternative to control malignancy [6]. There are various medicinal plants reported to have anticancer as well as anti-inflammatory activity in the Ayurvedic system of medicine. *Solanum nigrum* is one of them.

*S. nigrum* (Kakamachi) commonly known as black nightshade [7,8] usually grows as a weed in moist habitats in different kinds of soils, including dry, stony, shallow, or deep soils and can be cultivated in tropical and subtropical agro-climatic regions by sowing the seeds during April-May in well-fertilized nursery beds. Description of *S. nigrum* is found in all Ayurvedic literature. This drug was explained the first time in Vedic Granthas and was also used in Samhita period, especially in the form of Shakdravya (vegetable). *S. nigrum* crude extracts, as well as its purified compounds, have been used in various animal models such as rat, mice, and chick.

Both the crude extracts and isolated components of *S. nigrum* possess antiproliferative activity on various cancer cell lines. Crude extract is usually prepared with dried berries but can also be prepared from the whole plant. The antiproliferative activities of the crude organic extract and isolated compounds were studied on tumor cell lines of liver (HepG2), colon (HT29 and HCT 116), breast (MCF-7), and cervical (U14 and HeLa) [7].

Looking to the paucity of systemic work with regard to the potential of *S. nigrum* rat model in treating hepatocellular carcinoma (HCC), the present research work was carried out with the following objectives:

1. To prepare the aqueous and alcoholic extracts of *S. nigrum*
2. To study the effect of extracts of *S. nigrum* on gross and histopathology of the liver in NDEA-induced HCC in Wistar rats
3. To study the effect of extracts of *S. nigrum* on immunohistochemistry of the liver in NDEA-induced HCC in Wistar rats.

## Materials and Methods

### Ethical approval

The protocol of the experiment was approved by the Institutional Animal Ethics Committee with approval number 211/VCM/2015 approved by Letter No. AAU/GVC/CPCSEA-IAEC/129/2015, College of Veterinary Sciences and Animal Husbandry, Anand, Gujarat, and protocols were followed according to the guidelines of Committee for the Purpose of Control and Supervision of Experiments on Animals.

### Collection of plant materials

Plant of *S. nigrum* was procured from the field and was identified by Professor and Head, Department of Genetics and Plant Breeding, B. A. College of Agriculture, Anand Agricultural University, Anand.

### Acute toxicity testing of plant extracts

The acute oral toxicity study was carried out as per the Organization for Economic Cooperation and Development Guideline No. 423. Wistar rats were taken for the study and dosed once with 2000 mg/kg, orally. The treated rats were monitored for 24 h and up to 14 days for general clinical signs and symptoms, as well as mortality. It was observed that the aqueous and alcoholic extracts of *S. nigrum* have no toxic effect on rats even at 2000 mg/kg doses, respectively.

### Preparation of aqueous and alcoholic extract of S. nigrum

Leaves of *S. nigrum* were taken and dried under shade, then powdered by a mechanical grinder, sieved (Sieve No: 10/44), and stored in airtight containers. Exactly 100 g of coarse powdered material of *S. nigrum* was successfully extracted in Soxhlet extractor with water and methanol for aqueous and alcoholic extract, respectively. Extracts so obtained were decanted in a beaker and then concentrated to one-sixth of total volume in a water bath. The extracts were preserved in the refrigerator.

### Experimental animals

This study was conducted on 82 healthy adult male Wistar rats. Healthy adult rats of 6-8 weeks of age were procured from the Animal Research Facility, Zydus Research Center, Moraiya, Ahmedabad, Gujarat, India. Animals were acclimatized for 1 week before grouping and initiation of the experiment. All the rats were housed in polypropylene cages at Laboratory Animal House Facility in an environmentally controlled room with 22 ±3°C temperature and 30-70% humidity. Light/dark cycles of 12/12 h were maintained throughout the experimental period. All necessary managemental procedures were adopted to keep the rats free from stress.

### Induction of HCC using NDEA

The experimental hepatocarcinogenesis was induced using NDEA (Sigma Chemical Company, St. Louis, MO, USA). DEN is the most important environmental carcinogen among nitrosamines and primarily induces tumors of liver [9].

HCC was induced inGroup II, IV, V, VI, VII, and X rats by administering a single intraperitoneal injection of NDEA at a dose concentration of 200 mg/kg body weight mixed in saline. 2 weeks after administration of NDEA, 0.05% of phenobarbitone was administered by dissolving in drinking water up to 16 weeks to promote HCC [10].

### Experimental design

**Figure-1:**
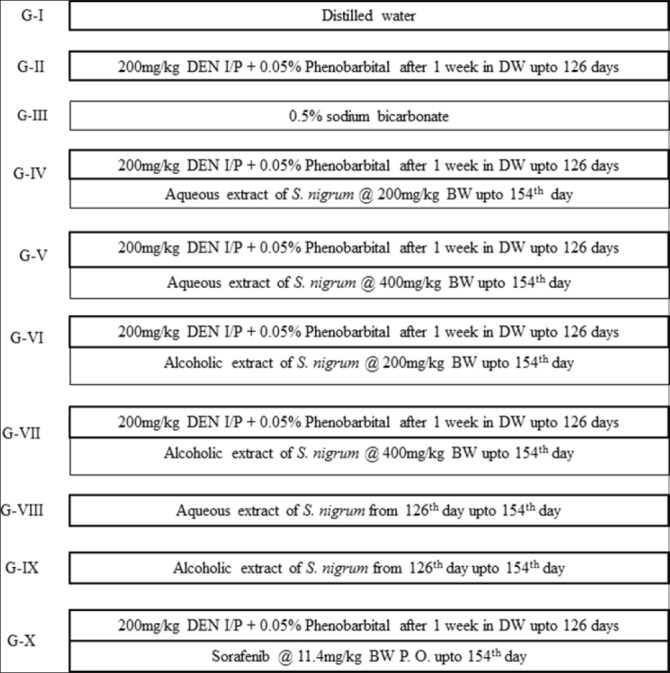
Schematic representation of experimental design.

Rats were selected randomly and divided into 10 groups (Groups - I-X) each group comprised of eight animals except Group II which contained10 animals (Figure-1). Since Group II is HCC control group there is chance of mortality during the experiment leading to decrease in the number of samples for statistical analysis. So we kept 2 extra rats so if there is death of 2 rats, still 8 rats will be available to take samples which will match other groups which has 8 rats. All the rats were numbered group wise and individually. Group I served as normal control consisted of healthy animals. HCC was induced in rats of Groups II, IV, V, VI, VII, and X using NDEA as inducing agent followed by phenobarbitone as a promoter. Group II animals were kept untreated. After a diagnosis of HCC in rats, Group IV and V rats were treated with aqueous extract of leaves of *S. nigrum*. Similarly, Group VI and VII rats were treated with alcoholic extract. Group X rats were treated with reference drug (sorafenib). Group VIII and IX animals were kept as aqueous and alcoholic extract control, respectively, for studying the effect of the same on normal rats.

### Gross and histopathological study

Gross and histopathological study of liver, spleen, and kidney were carried out after necropsy of Wistar rats. After the experimental period, animals were fasted overnight and sacrificed by cervical dislocation under anesthesiafollowing Animal Ethical Guidelines. Immediately after killing, liver, kidney, spleen, and heart were rapidly excised. Immediately after excising liver, it was washed in ice-cold isotonic saline, blotted to dryness, observed for the presence of hyperplastic nodules, and then weighed by digital weighing machine.

All tissue samples were collected in 10% formalin. Formalin-fixed tissues were processed by paraffin wax embedding method of tissue sectioning. Sections were cut at 5-6 µ thickness and were stained with hematoxylin and eosin (H and E) stain [11]. The H and E stained slides were observed under microscope and lesions were recorded.

### Immunohistochemistry

For immunohistochemistry, 4 µm sections of formalin-fixed paraffin--embedded specimens were cut and mounted on poly-L-lysine coated slides. The tissue sections were deparaffinized and rehydrated in graded ethanol. Sections were then rinsed and placed in a pressure cooker containing boiled citrate antigen retrieval buffer, pH 6 (Sigma-Aldrich, USA). The lid of a pressure cooker was sealed, and pressure cooker was allowed to reach full pressure. When the pressure indicator valve rose to the highest position, the sections were incubated for 2 min. The pressure cooker was transferred to a sink and cold water was run over the lid until all of the pressure was released. The cooled slides were washed in distilled water and placed in Tris-buffered saline (TBS) pH 7.4 for 20 min. Endogenous peroxidase was blocked by 3% H_2_O_2_ for 30 min followed by blocking with serum-free protein block (Dako). After pre-treatment, the sections were incubated overnight at 4°C with anti-cleaved caspase-3 (Asp175; D3E9, Cell Signaling) at 1:250 dilution. Following primary antibody treatment, tissue section was washed in TBS. After which, the sections were incubated (1 h) at room temperature with EnVision rabbit/mouse reagent conjugated to peroxidase (Dako REAL™ Envision™ HRP; Dako, Denmark). The sections were subsequently washed 4 times in TBS and developed with 3,3’-diaminobenzidine (DAB) as chromogen. The sections were then counterstained with Mayer’s hematoxylin, dehydrated, cleared, and mounted.

### Statistical analysis

Data obtained were analyzed using the standard statistical procedure as described by Snedecor and Cochran [12] and were expressed as mean±standard error of mean using SPSS software.

## Results

Complete chronological postmortem examination of the rats was done at the end of the experiment. Tissues such as liver, kidney, and spleen were collected from all the rats of different groups during necropsy for the macroscopic and microscopic examination. No gross and microscopic lesions were noted in kidney and spleen of rats from different groups indicating no action of any chemicals and drugs used in this experiment on those organs during the experiment.

**Figure-2:**
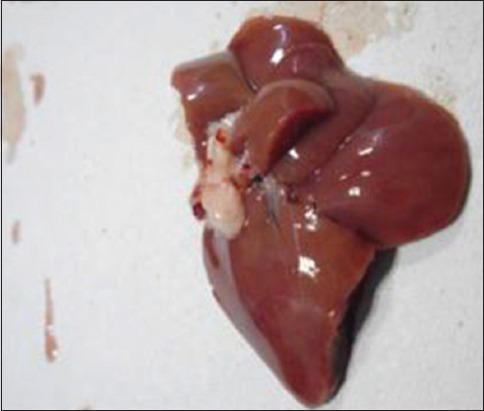
Normal liver from Group I rat.

**Figure-3:**
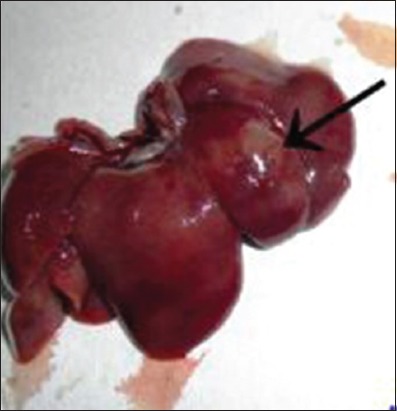
Liver of Group II rat showing development of nodule.

**Figure-4:**
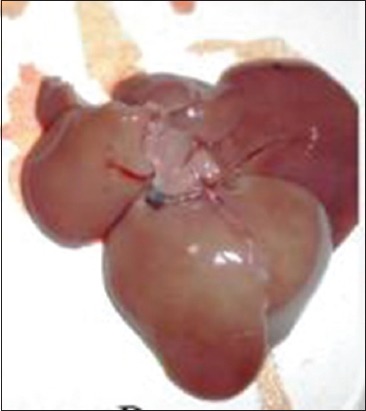
Group II rat showing pale appearance.

The liver was examined for gross abnormalities during necropsy in all groups. In the Group I, the liver was found to be normal (Figure-2). As expected, no gross abnormalities were observed. In the Group II, diffused white circular lesions of necrotic foci with pale appearance were observed on the liver (Figure-3) of one animal. Another animal in Group II showed the development of white nodules (Figure-4). Liver of animals in Group IV, V, VI, and VII were found normal in appearance after the treatment with aqueous and alcoholic extract of *S. nigrum*. Liver of rats from Group III, VIII, and IX showed no gross lesions indicating that there is no effect of vehicle used in the experiment and aqueous and alcoholic extracts of *S. nigrum*.

**Figure-5:**
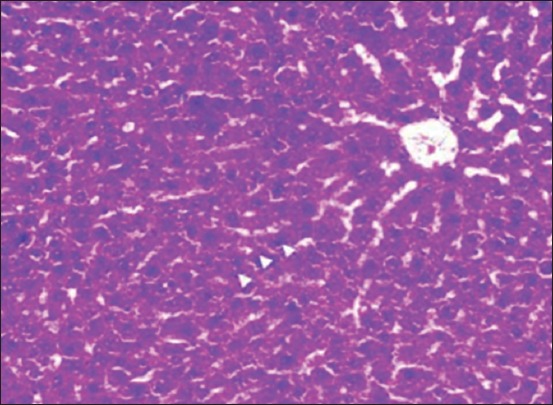
Section of liver from Group I rat showing anastomosing plates of hepatocytes radiated from the central vein toward the periphery of the lobule.

Histopathological examination of liver sections of normal control group (Group I) stained with H and E showed classical hepatic lobules. Each lobule showed anastomosing plates of hepatocytes radiating from the central vein toward the periphery of the lobule (Figure-5). The liver sinusoids were seen in between the adjacent plates. Kupffer cells were also seen associated with the sinusoidal lining cells. Liver sections of Groups VIII and IX which were administered with extract without the induction of HCC also showed the features of normal liver indicating that there is no adverse effect of the extract on the liver.

**Figure-6:**
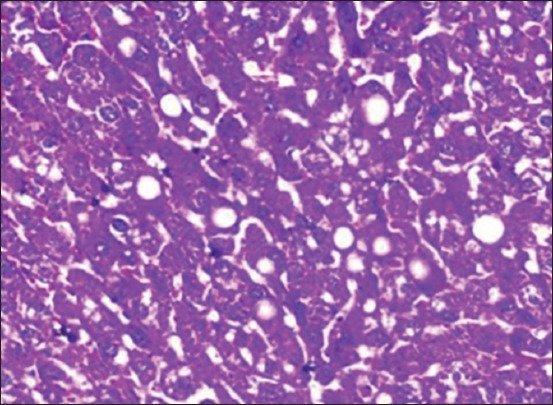
Section of liver from Group II rat showing fatty degeneration, necrosis, distortion, and condensation of nuclei before treatment.

In contrast, the sections of the liver from HCC control group (Group II) showed loss of lobular architecture. It also evidenced the features of necrosis and fatty change (Figure-6). Enlarged and darkened nuclei with variable size and dilatation of hepatic sinusoids with Kupffer cell hyperplasia were also observed. It also revealed the dilatation and proliferation of bile duct and intranuclear vacuoles and also showed the presence of more than one nucleolus. In NDEA treated (Group II, IV, V, VI, VII, and X), all rats showed neoplastic changes in liver section when compared with untreated control (Group I).

**Figure-7:**
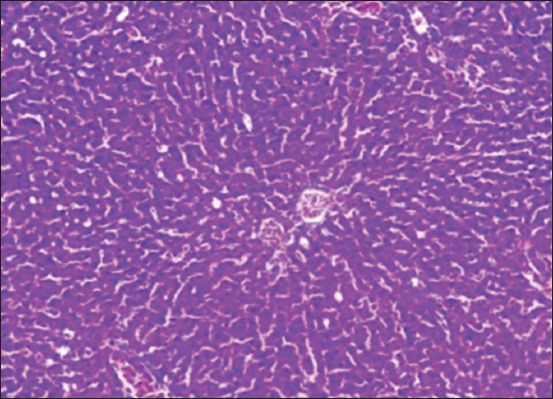
Section of liver from Group VII rat showing marked rejuvenation of hepatic architecture.

**Figure-8:**
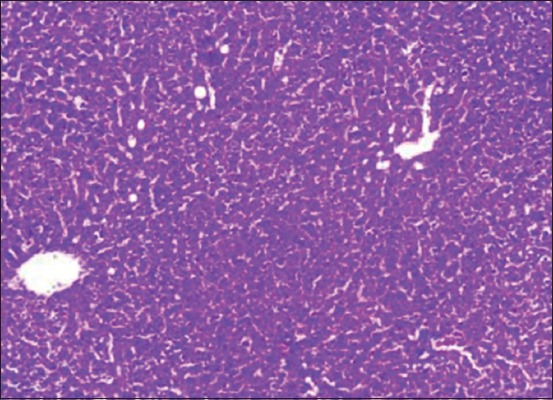
Section of liver from Group V rat showing reduction in the fatty degeneration.

Anticancerous property of *S. nigrum* was evidenced in the present study and was in accordance with the dose administered. Aqueous and alcoholic extract of *S. nigrum* to NDEA-treated rats reduced the severity of lesions in liver. Group treated with higher dose of extract after the induction of HCC (Groups V and VII) showed reduced degeneration and distortion of hepatocytes and marked rejuvenation of hepatic architecture (Figure-7) with restoration of bile duct histology. It also showed less fatty changes (Figure-8) and minimal pleomorphism compared to HCC control (Group II) rats. Group X rats which were administered with allopathic drug sorafenib were also examined for the comparison of the efficacy of our target Ayurvedic preparation. It revealed the less severe pathological changes compared to HCC control group. The liver sections of Group X revealed less fatty changes, less disarrangement, minimal pleomorphism, vacuolation, and degeneration of hepatocytes, and the result of this experiment reveals that there is no much difference between the extract and sorafenib-administered group. Liver of normal animals treated with 0.5% sodium bicarbonate (Group III) and aqueous and alcoholic extract (Groups VIII and IX) also showed normal architecture, indicating the non-toxic effect of 0.5% sodium bicarbonate and *S. nigrum* extract.

Liver sections of Wistar rats of different groups were subjected to immunohistochemical analysis at the end of the experimental period. The paraffin-embedded specimens were processed as per the standard procedure to analyze the immunoreactivity of cleaved caspase-3 in the liver sections to know the extent of apoptosis in the different groups of the experimental study.

**Figure-9:**
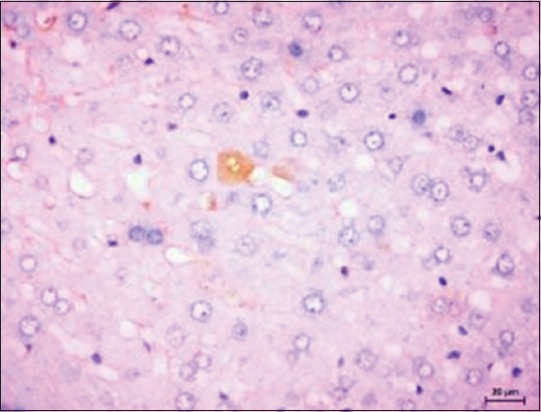
Hepatocytes from Group I rat showing immunoreactivity for cleaved caspase-3. Immunoperoxidase staining, 3,3’-diaminobenzidine chromogen, Mayer’s hematoxylin counterstain.

**Figure-10:**
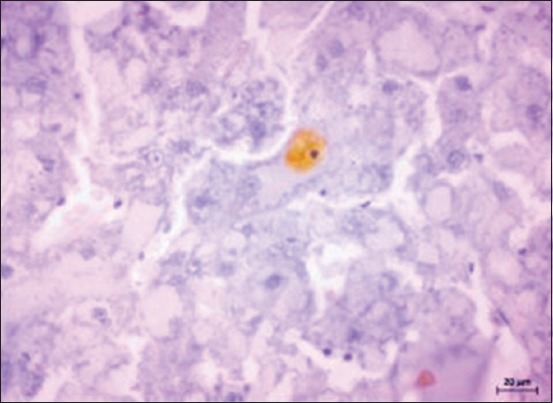
Section of liver from Group II rat showing rarely few hepatocytes showing immunoreactivity for cleaved caspase-3. Immunoperoxidase staining, 3,3’-diaminobenzidine chromogen, Mayer’s hematoxylin counterstain.

Immunohistochemistry was used for the detection of caspase-3-positive cells in liver samples. Immunohistochemical analysis of liver sections of normal control group (Group I) counterstained with Mayer’s hematoxylin showed classical immunoreactivity to cleaved caspase-3 (Figure-9). In contrast, the liver sections of hepatic cancer-induced group showed immunoreactivity to rarely few hepatocytes (Figure-10), indicating the proliferation of hepatocytes and inhibition of apoptosis due to uncontrollable proliferation of cells in tumor condition.

**Figure-11:**
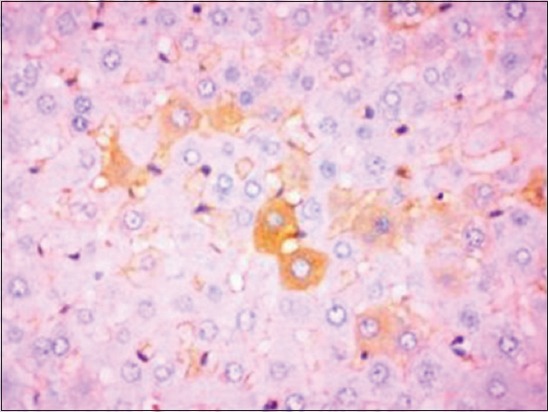
Section of liver from Group VII rat showing immunoreactivity for cleaved caspase-3 compared to normal control group. Immunoperoxidase staining, 3,3’-diaminobenzidine chromogen, Mayer’s hematoxylin counterstain.

**Figure-12:**
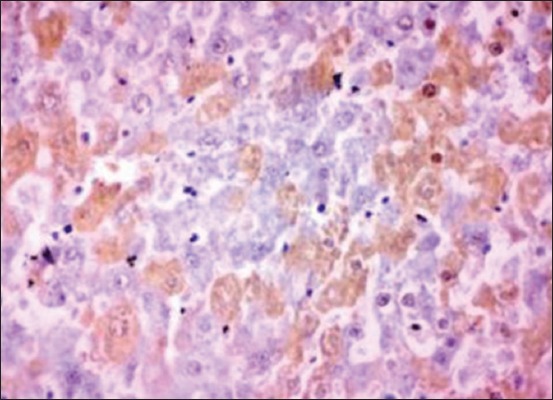
Section of liver from Group X rat showing many hepatocytes immunoreactivity for cleaved caspase-3. Immunoperoxidase staining, 3,3’-diaminobenzidine chromogen, Mayer’s hematoxylin counterstain.

Active caspase-3 foci were observed by immunohistochemistry in the liver sections of NDEA and *S. nigrum*-treated group as compared to Group II animals which were kept as a positive control group. Liver sections from Group VII showed increased immunoreactivity to cleaved caspase-3 compared to normal control group (Figure-11). Immunoreactivity of caspase-3 toward hepatocytes in the liver sections of group treated with standard drug sorafenib was analyzed to compare the efficacy of the target herbal medicine. Many hepatocytes showed immunoreactivity to cleaved caspase-3 (Figure-12). The present study revealed that the immunoreactivity of the hepatocytes of higher dose of alcoholic extract (400 mg/kg) is comparable to the group of rats treated with standard drug. This may be due to the cytotoxic and anticancerous efficacy of the herbal medicine toward NDEA-treated HCC.

## Discussion

The anticancerous activity of *S. nigrum* in NDEA-induced HCC in rats was investigated in the present study. Intraperitoneal injections of NDEA at 200 mg/kg body weight followed by administration of phenobarbital (0.05%) daily orally for 16 consecutive weeks showed loss of lobular architecture, features of necrosis and fatty change, enlarged and darkened nuclei with variable size, dilatation of hepatic sinusoids with Kupffer cell hyperplasia along with the dilatation and proliferation of bile duct, and intranuclear vacuoles and also showed the presence of more than one nucleolus. These observations suggest that chronic administration of NDEA develops HCC lesions. The histopathological observations were in consistent with the immunohistochemical changes in the liver where liver sections of hepatic cancer-induced group showed immunoreactivity to rarely few hepatocytes indicating the proliferation of hepatocytes and inhibition of apoptosis due to uncontrollable proliferation of cells in tumor condition.

Histopathological changes observed in the present study were in close conformity to the findings reported by Youssef *et al*. [13]. Similar findings were achieved by Mohammed *et al*. [14] who showed that treatment with NDEA leads to vacuolated hepatocytes with fatty change, dilated blood sinusoids, massive portal leukocytic infiltration, and disordered arrangement of dysplastic hepatocytes with typical hyperchromatic nuclei. Anticancerous property of alcoholic extract of *S. nigrum* was evidenced in that there was marked rejuvenation in the hepatic architecture after 28 days of treatment. It is because *S. nigrum* contains the substances, such as total alkaloid, steroid alkaloid, steroidal saponins, and glycoprotein, exhibiting antitumor activity as reported by Khattak *et al*. [15].

Tumor initiation, progression, and maintenance commonly involve alterations in apoptosis. Studies have shown that dysregulation of apoptosis is an important cause for the hepatocarcinoma formation. Accumulating data indicate that induction of apoptosis is a crucial event for chemoprevention of cancer by naturally occurring dietary agents [16]. Thus, we evaluated the changes of apoptotic-related factor and cleaved caspase-3. In the present study, there was a significant decrease in the expression of cleaved caspase-3 in hepatic cancer-induced group. This finding was in consistent with the study of Zhang *et al*. [17]. This is due to NDEA which inhibits the expression of caspase-3, but the administration of a higher dose of alcoholic extract of *S. nigrum* activated the expression of caspase-3. Similar findings by Moreira *et al*. [18] reported that active caspase-3-positive foci were observed by immunohistochemistry in livers of DEN plus melatonin groups compared to control animals. Immunoreactivity of caspase-3 toward hepatocytes in the liver sections of group treated with standard drug sorafenib was analyzed to compare the efficacy of the target herbal medicine. The present study revealed that the immunoreactivity of the hepatocytes treated with higher dose of alcoholic extract (400 mg/kg) is similar to the group of rats treated with standard drug indicating the anticancerous propertyof *S. nigrum*.

Similarly, Kannampalli *et al*. [19] studied on protective effect of *Cassia fistula* Linn. on DEN-induced HCC and oxidative stress in ethanol pretreated rats, where oral administration of the ethanolic leaf extract of *C. fistula* for 30 days to ethanol plus DEN-treated rats significantly improved the alterations in the markers of hepatotoxicity and oxidative stress, resulting in the reversal of most of the parameters studied, and were comparable to the standard hepatoprotective drug silymarin. The findings of the present study agree with the results of Joseph *et al*. [20] who evaluated the anticancerous efficacy of Ayurvedic milk extract of *Semecarpus anacardium* nuts on HCC in Wistar rats. Ayurvedic milk extract when administered showed a positive correlation with the action of doxorubicin.

## Conclusion

The present study shows that oral administration of aqueous and alcoholic extracts of *S. nigrum* at 200 and 400 mg/kg body weight for 28 days produce anticancerous effect against HCC in rats in a dose-dependent manner. However, based on the results obtained in this study, it seems that more detail studies on the mechanisms of anticancerous activity of this plant and its probable use in HCC are still to be investigated. Finally, we suggest that *S. nigrum* has a potential for newer therapeutic applications in the future.

## Authors’ Contributions

This study is the major component of the work toward the M. V. Sc. Thesis of the first authorGMA. SKR provided guidance during the entire experiment and corrected manuscript. GMA helped in the collection of the material and preparation of the manuscript. SHR helped in immunohistochemical analysis. DJG helped in the histopathological analysis of the liver sections. All authors have read and approved the final version of the manuscript.
